# Community Perspectives on Primary Prevention of Rheumatic Heart Disease in Uganda

**DOI:** 10.5334/gh.1094

**Published:** 2022-01-20

**Authors:** Hadija Nalubwama, Emma Ndagire, Rachel Sarnacki, Jenifer Atala, Andrea Beaton, Rosemary Kansiime, Rachel Mwima, Emmy Okello, David Watkins

**Affiliations:** 1 Uganda Heart Institute, Kampala, Uganda; 2 Children’s National Hospital, Washington DC, USA; 3 Cincinnati Children’s Hospital Medical Center, Cincinnati OH, USA; 4 Department of Pediatrics, University of Cincinnati College of Medicine, Cincinnati, OH, USA; 5 Division of General Internal Medicine, University of Washington, Seattle, WA, USA; 6 Department of Global Health, University of Washington, Seattle WA, USA

**Keywords:** Pharyngitis, rheumatic fever, rheumatic heart disease, Uganda, primary prevention

## Abstract

**Background:**

Untreated streptococcal pharyngitis is a precursor to rheumatic heart disease (RHD) and remains a significant public health issue in many countries. Understanding local determinants of treatment-seeking behaviors can help tailor RHD prevention programs.

**Objective:**

We sought to elicit perceptions of pharyngitis and related healthcare use in a range of communities in Uganda.

**Methods:**

We conducted six focus group discussions (FGD) in three districts that were representative of the country’s socioeconomic and cultural heterogenetity. Participants were recruited from six villages (two per district), and FGDs were audio recorded, transcribed and translated into English. Deductive and inductive analysis of the transcripts was done via open axial and sequential coding, which informed development of clusters, themes and subthemes. We extracted quotations from the transcripts to illustrate these themes.

**Results:**

We identified nine key themes in three major domains: knowledge and perception of pharyngits, treatment practices, and barriers to uptake of formal public-sector healthcare services. Community awareness and understanding of the consequences of pharyngitis were low. Stated barriers to care were usually systemic in nature and included low overall confidence in the healthcare system and substantial costs associated with transportation and medications.

**Conclusion:**

The FGDs identified several approaches to shape community perceptions of pharyngitis and improve utilization of interventions to prevent RHD. In Uganda, information-education-communication interventions probably need to be combined with structural interventions that make formal public-sector healthcare more accessible to at-risk populations.

## Introduction

Despite 70 years of clinical experience using penicillin to treat streptococcal pharyngitis and prevent rheumatic fever and rheumatic heart disease (RHD) [1,2], rates of untreated pharyngitis remain very high among school-aged children in most low- and middle-income countries [3]. In Uganda and other African countries, primary healthcare systems are under-resourced, and less than five percent of the population has access to pharyngitis care [4,5]. This lack of access partially explains why RHD remains a significant cause of cardiovascular mortality worldwide [6].

To address the persistence of RHD, the 71^st^ World Health Assembly adopted a Resolution mandating Member States to establish control programs that are built around improved pharyngitis care and delivery of secondary prophylaxis to individuals with a history of rheumatic fever or RHD [7]. To implement this Resolution, national ministries of health require information on local contextual factors that influence individuals’ decisions to seek care for pharyngitis and agree to the use of antibiotics when clinically indicated. Unfortunately, few such data exist: a recent systematic review of RHD research based in Uganda and Tanzania identified only one study related to pharyngitis [8,9]; however, this study only captured the perspectives of specialist clinicians and patients with RHD, not community members in the general population.

Recently, the Uganda Ministry of Health partnered with the Uganda Heart Institute, a publicly-funded, semi-autonomous research and clinical center in the capital city of Kampala, to develop a national strategy on RHD that would guide the local implementation of the World Health Assembly Resolution. This partnership led to a research initiative by the Uganda Heart Institute to better understand local rheumatic fever, RHD epidemiology and health service utilization patterns [4,10], and establish a baseline for monitoring the implementation of a national RHD program. The present study, which was part of this broader research initiative, sought to elicit community perceptions of pharyngitis and pharyngitis-related healthcare to inform the design of interventions that could increase the uptake of formal public-sector healthcare services for this condition and reduce the incidence of rheumatic fever and RHD.

## Methods

This study adheres to the COnsolidated criteria for REporting Qualitative research (COREQ) (*https://www.equator-network.org/reporting-guidelines/coreq/*). The appendix contains a completed version of the COREQ checklist for this manuscript.

### Study setting

Uganda is a country in eastern sub-Saharan Africa with a population of about 44 million, three-quarters of whom live in rural areas. In 2018, when data collection for this study began, net primary school enrollment was around 95%, and most adults were employed in the agriculture sector. About one-fifth of the population lived below the national poverty line. Current health expenditure in 2018 was US$ 43 per capita, 38% of which was paid for out-of-pocket [11]. Surveys have found a slight preference for private as compared to public sector healthcare, and around one in ten persons seek care at traditional practitioners, who often use a combination of herbal and spiritual treatments for ailments [12].

The above-mentioned RHD research initiative of the Uganda Heart Institute spanned several districts that were broadly representative of the economic, social, and demographic heterogeneity of the country. We conducted our focus group discussions in three of these districts – Lira, Mbarara and Wakiso. Lira district is in the Northern Region and is located about 340 kilometers from the capital. The district has the highest share of rural residents and has the lowest estimated gross domestic product (GDP) per capita of the three districts, at US$ 450 per person [13]. Mbarara district, located about 270 kilometers from Kampala, is an economic hub of the Western Region, with an estimated GDP per capita of US$ 1000 per person. Wakiso district, located in the Central Region and adjacent to Kampala, is a largely urban district and is the wealthiest in the country (estimated GDP per capita US$ 3300 per person). Literacy rates among adult females vary from 48% in the Northern Region to 70% in the Western Region and 78% in the Central Region [14].

### Research team

The focus group discussions were primarily facilitated by one author (HN) who is a public health officer with 8 years of experience (at the time of this study) conducting qualitative research. Three clinical research nurses (JA, RK, RM) with prior experience in qualitative research assisted the primary facilitator. All four facilitators identified as female, and at least one of the facilitators lived and worked in each of the districts in which the discussions were convened and was fluent in the local language. Research participants generally did not have a prior relationship with the study team.

### Study design and procedures

#### Overview and orientation

The objective of this study was to develop a ‘micro-level’ working theory of health behavior related to pharyngitis in Uganda. We viewed the focus group approach as the most appropriate method for assessing community-level perceptions of pharyngitis and general health behavior patterns. We used an exploratory approach to collect the data and did an initial inductive coding exercise to look for major themes. After review of these emerging themes, we switched to a confirmatory/deductive approach to look at variations in themes by location and setting and map the themes to a published model of health behavior (see below). Our study therefore blended interpretivist and positivist orientations.

#### Participant selection

Because we sought to understand attitudes and behaviors in the general population, we collaborated with village health teams (VHTs) to identify eligible participants. VHT members are residents of local communities who are appointed to serve as communities’ primary contact with the healthcare system. Before data were collected, we obtained approval from the district health offices to conduct this research and to partner with the VHTs.

Sampling occurred in two stages. In the first stage, we identified six villages in total from the three districts, with one urban and one rural village sampled in each district. The individual villages were randomly sampled from village lists provided to us by local authorities; we purposively selected two sub counties/parishes in each district based on their urban and rural settlement characteristics. The VHTs for these selected villages were contacted and oriented to the study. In the second stage, VHTs were trained by a member of the research staff and provided with scripted information to share during the recruitment process. The scripted information included the title and purpose of the study, evidence of approval from the district health officer, basic information on the study procedures, and assurance that participant data would remain anonymous.

The VHTs then walked door-to-door in their community and enrolled the first 10–12 individuals (one per household) in each district who were willing to participate and who met eligibility criteria, which we defined as (1) being over age 18, (2) having heard of sore throat, and (3) being able to provide informed consent. (While the FGDs were about pharyngitis among children, having one’s own child was not a part of the eligibility criteria.) Only those who were able to give written informed consent were enrolled in the study.

#### Data collection

Participant recruitment and data collection occurred between December 2018 and November 2019. The number of participants in a group ranged from 6–10. No enrolled individuals withdrew from the study.

FGD were convened in private community settings, such as churches or school grounds, that were chosen by the participants. Beyond the recruitment script, no additional information about the research team, including the facilitator, was provided unless requested by participants. Discussions were conducted in the participants’ preferred language, and at least two facilitators were present for all FGDs, with one facilitating the discussion and another assisting with notetaking and logistics. In keeping with the exploratory nature of the data collection, the primary facilitator used a semi-structured discussion guide (see appendix) that was roughly modeled after the ‘eight questions’ approach developed by medical anthropologist Arthur Kleinman; this approach has been adapted for other diseases and validated in the East African context [15].

All FGDs were audio-recorded with the permission of the group members, and identities were kept anonymous through the numbering of participants. Field notes were taken after the discussions using a semi-structured approach (see appendix). No one else was present for the discussions except for the researchers and participants. Each FGD lasted around 60–90 minutes, and participants were reimbursed for their time and travel costs with a stipend of 20,000 Ugandan shillings (about US$ 5.5). An interim analysis of field notes and recordings from the six FGDs suggested thematic saturation, so no additional FGDs were undertaken.

### Data analysis

Audio recordings were transcribed and translated into English for analysis. Transcript management and coding were done in Atlas.ti, version 8.4.25 and in Microsoft Word. Three team members (HN, EN and RS) participated in the coding and analysis. Before coding was attempted, the analysts read the transcripts several times to ensure familiarity with the data.

Two phases of analysis were done. The first (inductive) used a fully grounded-theory approach. Open, axial, and sequential coding procedures were used to develop clusters, themes, and subthemes. Each transcript was coded independently by at least two team members, one of whom did not participate in the original FGDs. The coders compared codes they had developed independently and refined their list of codes until consensus was achieved and a final code book developed. The appendix includes the code book with the list of codes used in analysis.

The second phase (deductive) sought to describe the variation in themes across relevant population strata, under the hypothesis that substantial variation would be found across urban-rural divides and across districts, and to map the findings to a health behavior model. We searched the literature for published models of health behavior and selected a model described by Fishbein and Yzer that was particularly useful for understanding ambulatory and preventive care-related behaviors [16].

The original study design had specified that we would undertake member-checking activities to add analytic rigor. However, this stage of our work was put on hold in March 2020 by the Covid-19 pandemic and a resulting suspension in nearly all human subjects research in Uganda. We decided to proceed with the analysis and write-up of the study to produce a set of findings that would influence the design of Uganda’s emerging national RHD strategy and time-sensitive research proposals. Consequently, no repeat FGDs or follow-up interviews were undertaken after the analysis was done.

### Ethics statement

The study was approved by the institutional review boards of Makerere University School of Medicine in Uganda (REC RF 2018-082), and the University of Washington in the United States (STUDY00002855). We sought clearance from Uganda National Council for Science and Technology (SS 5081) and permission to conduct the study from the relevant health authorities in all districts.

## Results

A total of 49 individuals participated in the six focus group discussions. Participants’ ages ranged 18 to 55 years, and nearly all were females. Four in ten respondents were unemployed, with the rest primarily engaged in farming or other small businesses. Six in ten had only attained primary education or secondary education; one in ten had no formal education. Three in four respondents were married.

### Thematic domains

The inductive phase of our analysis generated nine unique themes that we grouped into three domains: (1) knowledge and perception of pharyngitis, (2) treatment practices and (3) barriers to uptake of ‘formal public-sector healthcare services’ (FPHS). The appendix outlines these themes and presents a hierarchy of their importance, including mention of minor themes and diverse or atypical cases that contrasted with the major themes.

#### Domain 1: knowledge and perception of pharyngitis

In nearly all FGDs, most participants were familiar with sore throat but had never heard of the group A streptococcus bacterium and therefore did not identify this as a cause of sore throat. Poor hygiene, allergies, spirituality, and bad weather were the most frequently listed causes:

‘A child can get that sore throat through eating dirty things.’ *Respondent 2, Lira (urban)*

‘They always tell me it is an allergy or that it is the rainy season and that the weather is just bad.’ *Respondent 9, Mbarara (urban)*

‘We don’t know [what causes sore throat], we are also asking ourselves.’ *Multiple respondents, Wakiso (urban)*

‘The baby [child] dreams at night and the following day the baby [child] has giduan (sore throat) so we are believing that it has something to do with evil.’ *Respondent 9, Lira (rural)*

Despite their uncertainty about its cause, it was widely believed that pharyngitis could be spread person to person. Participants perceived it to be a serious health condition that was prevalent in communities, especially in rural villages:

‘I think maybe it moves with air sometimes because if my [child] is suffering from it, after two to three days you will find another [child] at another home’ *Respondent 9, Lira (Rural)*

‘I have noticed that this illness is common in our village. I don’t know what you as doctors are going to do to treat it’ *Respondent 9, Mbarara (rural)*

‘[Sore throat] is serious just like malaria. Just like when you don’t treat malaria, the [child] dies’ *Respondent 1, Lira (rural)*

Participants observed that pharyngitis often recurred, and they were concerned about its interference with eating and drinking. A child’s failure to eat or drink was identified as a major symptom that parents relied on to suspect the presence of pharyngitis in children and to determine its seriousness.

‘However much you try to feed him [the child], he couldn’t swallow. That is the sign that your child is doing badly because everything you give him, he would not eat it. As a parent you develop fear.’ *Respondent 2, Wakiso (rural)*

In addition, they noted that sore throat can progress to other conditions (such as cancer) or otherwise lead to bad outcomes or death:

‘What worries me is that sore throat can cause cancer.’ Respondent 7 Mbarara (urban)

However, none of the participants mentioned heart disease (including RHD) as being a consequence of pharyngitis.

#### Domain 2: Treatment practices

In this study, we defined FPHS as healthcare offered at public or private health facilities and that usually follows contemporary, Western-based medical practices and makes use of pharmaceuticals. We defined ‘traditional medicine’ (TM) as the treatment of illness with a combination of herbs, local physical interventions, other natural remedies, and spirituality. TM is usually provided by a community spiritual leader or healer.

One type of TM/physical intervention that is unique to certain communities in Uganda is a procedure called ‘local tonsillectomy’ or ‘crude tonsillectomy’ [17]. This procedure is done by TM practitioners and involves direct, intentional trauma to inflamed tonsils, with the intensity of the trauma varying from a minor abrasion to full excision, depending on the practitioner. Beliefs about the efficacy of crude tonsillectomy vary widely within communities, and practitioners of FPHS typically look unfavorably on the procedure due to the high infection risks involved, leading to stigma.

Community members reported a range of treatment practices in their districts. While some do not seek treatment for sore throat at all, most seek some form of care through TM, FPHS, or frequently, a combination of the two. Participants reported that TM offered convenience, reduced costs, and faster improvement in symptoms, which is why many in their communities used these therapies:

‘I took the [child] to the hospital, [and] I found that there [was] no change. I came back to… [the healer]; she removed [the tonsil], and I took the [child] back to the hospital.’ *Respondent 9, Lira (rural)*

‘My child had [sore throat] once, but I used [*Chenopodiaceous* herb]… I mixed it with [black salt]… it healed.’ *Respondent 4, Wakiso (rural)*

‘When you go to the [healer], even if you don’t have the money, she just helps you. She just serves and tells you that when you get money you [can] bring it to her.’ *Respondent 2, Lira (rural)*

Some reported that traditional remedies are preferred over FPHS because of negative perceptions and fear of FPHS:

‘When the [child] is suffering from [sore throat], … the drugs from the hospital… can kill the [child].’ *Respondent 4, Lira (urban)*

Others indicated a preference for FPHS, citing religion, confidence in FPHS and/or painful or negative experiences with traditional healers, including the death of a child after crude tonsillectomy:

‘Whenever my children get sore throat, I always take them to the hospital… My first child had a sore throat; I went for the local tonsillectomy… [and] my child died there.’ *Respondent 5, Lira (urban)*

Participants indicated that people do not often complete all prescribed treatments, whether TM or FPHS. The most common reason for stopping any type of treatment was an improvement in a child’s symptoms. Other reasons included forgetting and difficulties in swallowing medicines:

‘When you give [the child] medicine and [they are] fine in a day, you stop giving the treatment… [even though the] doctors advise [taking treatments for several] days. Sometimes we go to the clinic and only buy medicine that we can afford.’ *Respondent 2, Mbarara (urban)*

‘Sometimes… the doctor tells you to give… medicine for five days; maybe I give [it] in the morning, but in the afternoon, I forget.’ *Respondent 7, Lira (rural)*

#### Domain 3: Barriers to uptake of FPHS

Many participants reported a belief that FPHS could be safer and more effective than TM. However, they cited numerous barriers, which we grouped into two clusters: high cost of care and low quality of care.

Participants described substantial direct costs, including out-of-pocket payments for care and non-medical costs like transportation, as well as indirect costs, including lost time off work (parents/caregivers) or school (sick children). Many participants, especially those in rural communities, reported having to travel long distances to health facilities at great personal expense. Compounding this problem, the availability of free medicines at public health facilities is not guaranteed. When these medicines are not available, patients are forced to go to private health facilities and buy their medications at high prices:

‘The problem we have is lack of drugs in government hospitals. You might come to the hospital, [and there are] doctors but there are no drugs.’ *Respondent 1, Wakiso (urban)*

‘You may have to go [to the health facility] three times to succeed [at getting medicines].’ *Respondent 1, Lira (rural)*

‘[School children] are affected because when [they have] that illness they [will] not go to school’ *Respondent 2, Wakiso (urban)*

‘When you come to the public clinic… you can spend the whole day.’ *Respondent 3, Lira (urban)*

In some households there were interpersonal costs to sore throat treatment. Several participants cited the lack of support from their spouses:

‘It’s only… the mothers who care about [sore throat]. The men look at it as a normal thing.’ *Respondent 3, Wakiso (urban)*

Participants also reported hostility or poor treatment that they or other community members received when seeking FPHS, which made them less likely to seek care at such a facility in the future. Individuals who first received TM and then sought FPHS were sometimes mistreated by FPHS practitioners:

‘Sometimes [if you say] that you went and [had a crude tonsillectomy], [the health workers]… tell you, “we are not going to work on that kid.”’ *Respondent 3, Lira (urban)*

In addition, participants reported that FPHS practitioners did not always confirm the diagnosis, and some gave malaria treatment presumptively:

‘In most cases when you go to hospital, [the health workers] will… [just] say it is malaria… they can never confirm the [diagnosis].’ *Respondent 5, Lira (rural)*

### Variation in themes

**Figure 1:**
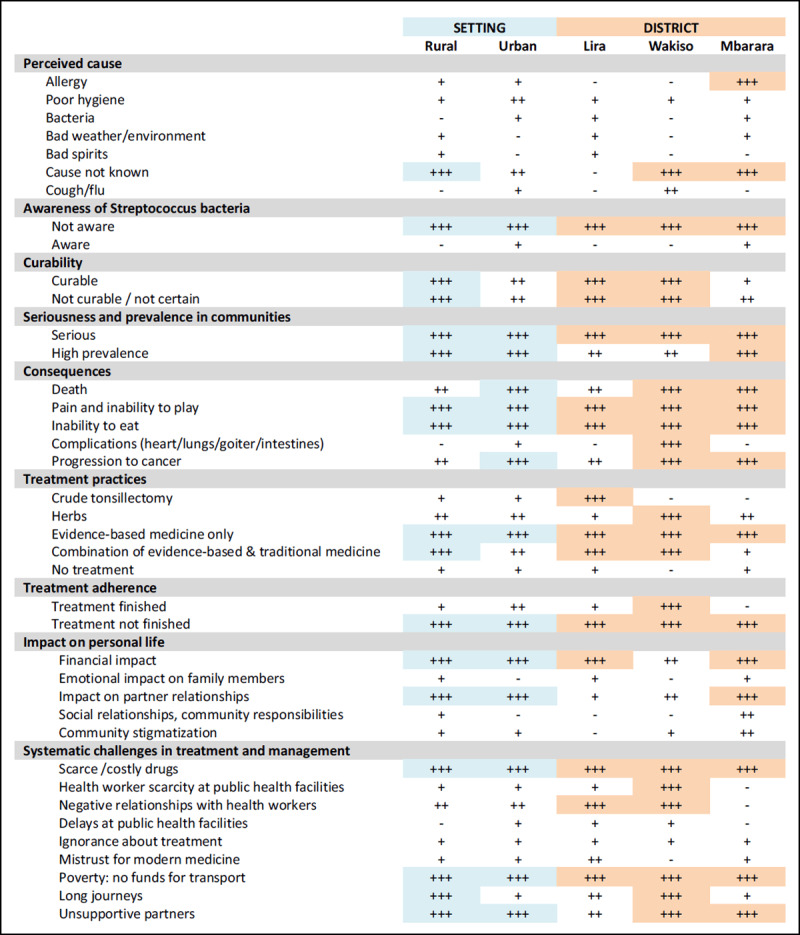
**Data display matrix showing the relative frequency of themes by setting and district.** Symbols indicate: – not noted in any transcripts; + noted in very few transcripts; ++ noted in significant number of transcripts; +++ noted in nearly all transcripts.

The deductive phase of our analysis focused first on variations in themes across settings (rural vs. urban) and districts. We hypothesized that there would be considerable differences because of cultural and socioeconomic differences. To test this hypothesis, we did a comparison of the relative frequency of themes across these strata. ***Figure 1*** summarizes the differences we found in themes between settings and across districts.

Between rural and urban settings, knowledge and perceptions about pharyngitis varied: rural participants more frequently stated that the cause of pharyngitis was not known; urban participants were generally more likely to describe serious consequences such as death and cancer. While treatment practices were similar, and most participants reported use of FPHS, urban participants were less likely to use a combination of FPHS and TM. The most notable difference between urban and rural settings regarding barriers to uptake of FPHS was that rural participants more frequently described long travel times to receive care.

Across the three districts, the main differences in pharyngitis knowledge and perceptions were that (1) participants in Mbarara were more likely to ascribe pharyngitis to allergies, (2) participants in Wakiso and Mbarara were more likely to state that the cause of pharyngitis is unknown, and (3) participants in Lira mentioned serious complications of pharyngitis, including death, considerably less frequently. Participants in Mbarara also cited a high frequency of community stigmatization around pharyngitis. Regarding treatment practices, the use of crude tonsillectomy was only mentioned by participants from Lira. Participants from Mbarara most frequently reported exclusive use of FPHS. Participants from Wakiso most frequently reported completing a full course of treatment. Participants from Mbarara more frequently noted impacts of pharyngitis on relationships with their partners.

Finally, barriers to uptake of FPHS across the three districts also varied. Whereas lack of medications at facilities and lack of funds for transportation were noted throughout the districts, participants from Wakiso more frequently reported a lack of health workers at public facilities. Participants in Lira and Wakiso described negative relationships between health workers and the community that was not described by participants in Mbarara. Long transportation times were most frequently mentioned in Wakiso, which is a geographically very large district.

### Link to health behavior model

Communications researchers Fishbein and Yzer noted that, around the year 2000, there were three theories used widely in health behavior research: (1) the health belief model, (2) social cognitive theory, and (3) the theory of reasoned action. They integrated the insights and constructs from these theories into one ‘integrated theoretical model’ that was designed to predict health behaviors based on a limited number of variables [16]. They theorized that health behaviors were determined primarily by intentions, skills and abilities, and lack of environmental constraints. Intentions, in turn, were directly determined by attitudes, perceived norms, and self-efficacy, and they were indirectly influenced by numerous ‘distal’ factors such as culture, personality traits, and media consumption, among others. Fishbein and Yzer argued that, by first characterizing the degree to which a particular health behavior in a particular group appears to be under the control of each of these factors, one could design an intervention to address the most important factors, maximizing the chance of engaging in the desired behavior (or not engaging in an undesirable behavior).

**Figure 2:**
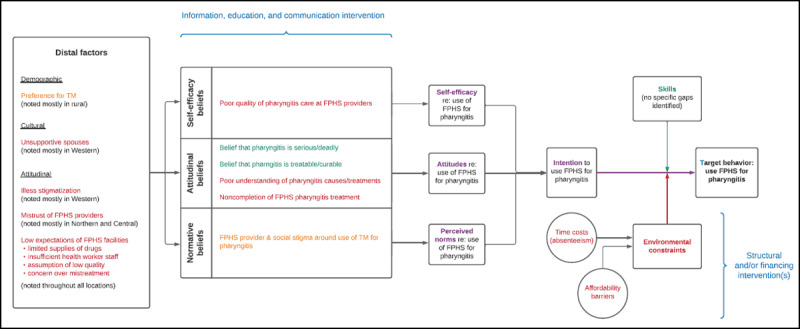
**Integrated conceptual model of health behavior related to pharyngitis treatment.** This figure was adapted from the integrated theoretical model proposed by Fishbein and Yzer [16], with themes from this study overlaid on the constructs in that model. Text in red denotes a barrier to the target behavior, use of FPHS for pharyngitis. Text in green denotes an enabler of the target behavior. Text in orange denotes a barrier that was only identified in some settings or districts. The text in blue shows how different types of health interventions could be designed to increase the chance of engaging in the target behavior. FPHS = formal public-sector healthcare services. TM = traditional medicine.

We went back to the codes and themes identified in the previous phase of our analysis and linked them to the constructs in Fishbein and Yzer’s integrated theoretical model (***Figure 2***). We drew three key insights from this exercise.

First, there are several opportunities to improve ‘intentions’ to seek FPHS for pharyngitis. Communications interventions could target community and individual knowledge about the causes and consequences of pharyngitis and in some settings address stigma around the condition. Complementary efforts to improve the quality of pharyngitis care delivered and, where relevant, address stigmatization by health workers, might also enhance intentions.

Second, pharyngitis-related beliefs must be considered in the context of distal attitudinal factors. Mistrust of FPHS in general is a challenge that applies to a minority of Ugandans, more commonly those living in rural settings. On the other hand, most Ugandans have low expectations of FPHS, driven by previous experiences with long wait times, staffing shortages, medication stock outs, and, in some cases, concern over misdiagnosis or mistreatment.

Third, enhanced intention is probably unlikely to change behavior in the absence of efforts by health officials to reduce environmental constraints, including the time required to seek care and the affordability of transportation and medications.

## Discussion

This qualitative study on community perceptions of pharyngitis care in Uganda adds to a small literature on health behavior in relation to rheumatic fever and RHD prevention services. Using quantitative methods, studies in Ethiopia [18] and Cameroon [19] found low levels of population awareness regarding the link between pharyngitis and RHD. To our knowledge, ours is the first qualitative study to look at pharyngitis-related attitudes and behaviors in a country where RHD remains endemic. Our study goes beyond a description of this problem to look more deeply at a range of local factors that could be addressed to enhance the use of FPHS for pharyngitis in Uganda.

Although we found low levels of knowledge about pharyngitis and the importance of receiving FPHS, we also found that much of the ‘failure’ to engage in care appeared to be due to distal factors, such as a lack of trust in the public healthcare system in general, and environmental constraints, like time costs (especially for rural communities) and financial costs, principally medication and transportation costs. A previous study on rheumatic fever treatment adherence in Uganda found very similar themes to our study [20]. One implication of this research is that, on their own, communication interventions that seek to change intentions to seek care would have a limited impact. Efforts to enhance use of FPHS for pharyngitis need to be tied to efforts to improve the overall quality of and trust in the primary healthcare system.

At the same time, information-education-communication interventions have been shown to be effective at mobilizing communities to increase the use of RHD preventive services [21,22], and in Uganda, such interventions increased referrals for rheumatic fever evaluation [10,23]. In the Ugandan context, a communication intervention could have three major components: (1) teach communities about the causes of pharyngitis and the effectiveness and safety of FPHS, (2) leverage pre-existing enabling beliefs (e.g., that pharyngitis is serious and potentially deadly) to spur patients and families to action in the face of potential mistrust in the healthcare system, and (3) train health workers on proper pharyngitis management, including proper use of antimicrobials, while encouraging respectful and compassionate care for vulnerable populations. Information-education-communication interventions would need to be designed to account for the relatively low adult literacy levels in the poorer parts of the country such as the Northern and Eastern Regions.

Removing environmental constraints to pharyngitis care is primarily the responsibility of the healthcare system. Concerns over the financial cost of care could be addressed by bolstering supplies of (free) essential antibiotics at public healthcare facilities to prevent patients from having to go to private pharmacies and pay for medications out-of-pocket. In addition, programs targeting transportation affordability among rural and poorer populations could be considered, including mobile phone-based reimbursements for healthcare-related transportation [24], and government programs to enhance bicycle ownership [25]. Again, these barriers are not unique to individuals with pharyngitis, so a coordinated, whole-of-healthcare-sector strategy would be desirable.

Another response to these environmental constraints would be to experiment with alternative pharyngitis care delivery models. School-based, nurse-led evaluation and treatment [26] could quickly achieve high population coverage—since nearly all Ugandan children attend primary school—and would obviate the need for a caregiver to take a child out of school to a clinic at their own expense. Alternatively, in very highly endemic settings and seasons, community health workers could be trained in ‘active case-finding,’ going door-to-door to find and treat cases of pharyngitis using oral antibiotics [5]. Finally, the frequent use of TM alone or in combination with FPHS underscores the need for local public health officials and planners to work with traditional practitioners to identify a balanced approach that allows all at-risk children the opportunity to receive timely care without undermining trust or autonomy. Targeted, culturally sensitive approaches would be needed to address the practice of crude tonsillectomy in northern Uganda (i.e., Lira district) [17].

The consistency of themes across the three districts and between urban and rural settings suggests that interventions could be developed by the national Ministry of Health and deployed with minimal local adaptation. Still, our focus groups uncovered some local contextual factors (***Figure 1***) that might help adapt generic intervention templates to specific regions or districts. For example, communication interventions deployed in the Western region and Northern region would need to pay particular attention to stigmatization of pharyngitis and to the risks of crude tonsillectomy, respectively. Interventions deployed in rural populations would need to include adequate health education on the causes and consequences of pharyngitis and the benefits of seeking care despite the costs.

Our study has several important limitations. Our sampling frame and approach was restricted to a few districts that may not capture all the cultural and socioeconomic variation in Uganda and therefore might miss some important issues that are specific, e.g., to the eastern region of the country. Prior studies have raised concerns regarding access to high-quality penicillin in African settings [27]. Our study was not designed to assess ‘supply-side’ barriers to use of FPHS, such as stockouts of antibiotics, but these barriers would need to be considered and remediated alongside other structural barriers to care. Additionally, because of Covid-19 we were unable to do member-checking to maximize the internal validity of our study. The focus group discussion approach is ideal for capturing community-level attitudes and behaviors but does not provide the rich, in-depth understanding of patient experiences with pharyngitis care that an in-depth interview approach would provide. Related to this, the Fishbein and Yzer model was primarily developed for use alongside individual-level quantitative datasets to aid in behavior prediction. Our non-standard use of this model was meant to generate a working theory of pharyngitis behavior to inform the design of interventions. Our working theory will need to be validated by prospective, quantitative data collection, e.g., during the pilot phases of complex interventions that includes information-education-communication components.

## Conclusions

This focus group discussion study conducted in three diverse districts in Uganda uncovered a range of opportunities to shape community perceptions of pharyngitis and improve utilization of FPHS to prevent rheumatic fever and RHD. While community awareness of the causes and consequences of pharyngitis was low, most of the stated barriers to care were systemic in nature, such as low level of confidence in the public healthcare system overall and substantial costs associated with transportation and medications. Our findings suggest a need to combine information-education-communication interventions with structural interventions that bring FPHS closer to where individuals live and study.

## Data Accessibility Statements

Redacted versions of the focus group discussion transcripts can be found at *https://doi.org/10.7910/DVN/UL38AK*.

## Additional File

The additional file for this article can be found as follows:

10.5334/gh.1094.s1 Appendix. Supplementary Appendix.
