# Supplementary material for: Community Perspectives on Primary Prevention of Rheumatic Heart Disease in Uganda
Source: Glob Heart. 2022 Jan 20;17(1):5. doi: 10.5334/gh.1094 (PMC8782090; doi:10.5334/gh.1094)
Supplement: Appendix. — Supplementary Appendix. [file gh-17-1-1094-s1.pdf]

SUPPLEMENTARY APPENDIX

Discussion Guide for community focus groups regarding sore throat in children)

Objective: Develop a local explanatory model of sore throat (for use in designing interventions)

Selection criteria: Non-related community members (all ages 18+, both sexes) living in urban and rural locations in the district

Instructions:

Turn on recorder.

Provide copies of informed consent forms to all participants; read the form aloud for those who can't read. Tape-record any verbal consents.

Use the guide below to address these topics/questions, but maintain a good flow of conversation above all else. Encourage all group members to participate in the discussion (and actively seek out those who seem reticent or introverted).

First, explain the ground rules and make sure everyone agrees with them:

- There are no right or wrong answers
- We want to know everyone's viewpoint, so everyone should contribute
- We need you to be frank; all opinions are important even if there are disagreements
- Treat others with respect whether or not you agree with them
- Please do not tell anyone outside this group about what was said here
- Your responses will not be linked back to your personal identity in any reports

Start by having everyone introduce themselves. (Facilitator and any other research staff should also introduce themselves and their roles.)

Q1. This is a discussion about sore throat in kids. Does anyone want to share a recent experience they had with a child who had a sore throat?

Q2. According to you, what are the reasons a child gets a sore throat?

- What do you think causes sore throat in kids?
- Why do you think sore throat starts when it does?

Q3. In your opinion/experience, what do you think sore throat does to children?

- How severe of a problem is sore throat?
- Do you think it will last a long time or improve quickly?
- What worries you most about sore throat? What worries children most?

Q4. What sorts of problems does sore throat cause in this community?

- How does it affect the children (e.g., time off school, need for medical care)
- How does it affect families of children who have sore throat?
- Does sore throat affect the larger community in any way? Do you see it as a big problem or not a big problem in this community?

Q5. What do people in this community do when their child gets a sore throat, if anything?

- Medicines from pharmacies?
- Go to traditional healers?
- Go to a health centre?
- Others?

Q6. What results do people in this community hope these treatments will provide?

- Medicines from pharmacies?
- Go to traditional healers?
- Go to a health centre?
- Others?

Q7. Has anyone heard of strep throat infection? If so, what have you heard about it? What do people say should be done treat it?

- Use of antibiotics
- Delay in receiving antibiotics
- Duration of antibiotic use

Q8. Do people in this community trust the health centres to provide treatment for sore throat?

- Factors that foster confidence in health system and encourage care-seeking
- Factors that dissuade from seeking care at health centres (e.g., bad past experiences, financial barriers, etc.)

Thank participants for their time. Turn off recorder.

Provide opportunity for them to give feedback and ask questions.

Provide them with cash.

Fill out field notes once they've left.

Field Notes

Setting of focus group discussion (geographic location/address and type of building/place):

Appearance and demeanor of group participants:

Demographic/socioeconomic composition (age groups, gender, occupations represented):

Notable behaviors or nonverbal cues (i.e., not captured in recording/transcript), presence of especially talkative or quiet participants (quantify both, if possible):

Any changes or adaptations of discussion topics, probes, etc. for this particular discussion?

Reflections:

- Did the discussion feel easy or difficult? Unnecessarily brief or lengthy?

- How did I perform during the interview? Was I successful in facilitating a balanced discussion where all members had opportunities to share their views?

- Do I note any potential biases or feelings of mine that came through during the interview and affected the conversation?

Code Book

Domain 1: Knowledge and perception of pharyngitis		
**Code group**	**Code Name**	**Code description and code qualification**
**Perceived cause**		
	Allergy	Any mention of pharyngitis cause that is related to an allergic reaction
	Poor hygiene	Any mention of pharyngitis cause that is related to any bacteria or poor hygiene practices among either the children or the people they live with
	Bacteria/germs	Any mention of pharyngitis cause that is related to any bacteria or poor hygiene practices among either the children or the people with whom they live with
	Bad weather/environment	Any mention of pharyngitis cause that is related to either the dirty environment someone stays in or weather conditions
	Bad spirits/spiritual	Any mention of pharyngitis cause that is related to either fate, luck or beliefs
	Cause not known	Any mention of pharyngitis cause not being known. This could either be the respondents or other people in the community not knowing
	Cough/flu	Any mention of Pharyngitis cause as either cough or flu or both
**Awareness of Streptococcus bacteria**		
	Not aware	Any mention of or indication of a lack of awareness about streptococcus bacteria
	Aware	Any mention of having awareness about streptococcus bacteria
**Curability**		
	Curable	Any mention of pharyngitis being a curable illness when treated
	Not curable / not certain	Any mention of pharyngitis being incurable or not being sure if it is curable
	Cure vs. Symptom Relief	
**Seriousness and prevalence in communities**		
	Serious	Any mention of pharyngitis as a serious disease (worthy of attention) to the health of the children
	High prevalence	Any mention of pharyngitis being common among children in their community or other communities (varying rates of prevalence noted)
	Spread among community	Any mention of pharyngitis being spread between community members (witnessed or feared)

**Consequences**		
	Death	Any mention of death being the likely effect from pharyngitis and cause of worry
	Pain and inability to play	Any mention of the child not being able to play and feeling pain
	Impact on eating or drinking	Any mention of the child not being able (or willing) to eat or drink as usual
	Complications to other organs	Any mention of perceived complications to other body organs/systems resulting from pharyngitis
	Progression to cancer	Any mention of cancer as a possible consequence of sore throat
**Domain 2: Treatment**		
**Treatment practices**		
	Traditional medicine<br><br>Crude tonsillectomy<br><br>Local Herbs	Any mention pharyngitis treatment practices in children where traditional medicine was used. This could be local herbs, crude tonsillectomy or any other natural or spiritual remedy<br><br>Any mention pharyngitis treatment practices in children where direct intentional trauma to was caused to inflamed tonsils without modern medicine. This could range from a minor abrasion to full excision<br><br>Any mention of pharyngitis treatment using local herbs without any modern medicine
	Evidence-based medicine only	Any mention of pharyngitis treatment where modern/western, evidence-based medicine was used as the only treatment method
	Combination of evidence-based & traditional medicine	Any mention of pharyngitis treatment where both modern and traditional medicine (herbs, local tonsillectomy) were used for treatment
	No treatment	Any mention of not giving any form of treatment (either traditional or evidence-based) to children with pharyngitis
**Treatment adherence**		
	Treatment completed	Any mention of completing the recommended pharyngitis treatment (injections, pills, clinical reviews, etc.) The treatment could be either in form of injections or pills.
	Treatment not completed	Any mention of not finishing the recommended pharyngitis treatment (injections, pills, clinical reviews, etc.) for any reason such as having symptom relief and forgetting to take the medicine
**Treatment impact**		
	Financial impact	Any mention of the effect of pharyngitis on income and expenses in a household with a sick child

	Emotional impact on family members	Any mention of anxiety, stress, fear, panic, etc. related to having children with pharyngitis
	Impact on partner relationships	Any mention of pharyngitis (or having a child with pharyngitis) negatively or positively impacting on person's relationship with their partner
	Social relationships, community responsibilities	Any mention of pharyngitis influencing people's engagement in community activities (school, social, financial, spiritual groups, gatherings, other activities, etc.)
	Community stigmatization	Any mention of being stigmatized (demeaned, teased, shunned by community members) due to pharyngitis
**Domain 3: Barriers to uptake of evidence-based medicine**		
**Systematic challenges in treatment and management**		
	Scarce drugs	Any mention of challenges related to finding recommended drugs for treatment of pharyngitis
	Health worker scarcity at public health facilities	Any mention of challenges related to finding health workers at public health facilities
	Negative relationships with health workers	Any mention of challenges (negative attitudes, actions, communications) related to relationships between health workers and patients and their families.
	Delays at public health facilities	Any mention of challenges related to the perceived time spent at health facilities being beyond what was ideal to patients
	Ignorance about treatment	Any mention of barriers related to not having knowledge about or not understanding pharyngitis management. This could involve knowledge on causes, disease seriousness, risk factors and treatment
	Mistrust of modern medicine	Any mention of perceived unreliability of modern medicine in treating pharyngitis in terms of patient recovery time, medicine effectiveness, diagnosis, etc.
	Lack of funds for transport or medication	Any mention of barriers related to the inability to pay for or raise money to access pharyngitis treatment (e.g., transport or medication).
	Long journeys	Any mention of long distances travelled to reach health facilities for diagnosis or treatment (and impact on school, work, finances, etc.)
	Unsupportive partners	Any mention of a barrier related to partner support

COREQ 32-ITEM CHECKLIST*

Item #	Guide questions/description	Reported on p. #
**Domain 1: Research team and reflexivity**		
1. Interviewer/facilitator	Which author/s conducted the interview?	4
2. Credentials	What were the researcher's credentials?	4
3. Occupation	What was their occupation at the time of the study?	4
4. Gender	Was the researcher male or female?	4
5. Experience and training	What experience or training did the researcher have?	4
6. Relationship with participants established	Was a relationship established prior to study commencement?	4
7. Participant knowledge of the interviewer	What did the participants know about the researcher?	4-5
8. Interviewer characteristics	What characteristics were reported about the inter viewer/facilitator?	4-5
**Domain 2: study design**		
9. Methodological orientation and Theory	What methodological orientation was stated to underpin the study?	4
10. Sampling	How were participants selected?	4
11. Method of approach	How were participants approached?	4-5
12. Sample size	How many participants were in the study?	6
13. Non-participation	How many people refused to participate or dropped out? Reasons?	5
14. Setting of data collection	Where was the data collected?	5
15. Presence of non-participants	Was anyone else present besides the participants and researchers?	5
16. Description of sample	What are the important characteristics of the sample?	6
17. Interview guide	Were questions, prompts, guides provided by the authors?	5
18. Repeat interviews	Were repeat interviews carried out?	6
19. Audio/visual recording	Did the research use audio or visual recording to collect the data?	5
20. Field notes	Were field notes made during and/or after the interview?	5

21. Duration	What was the duration of the interviews?	5
22. Data saturation	Was data saturation discussed?	5
23. Transcripts returned	Were transcripts returned to participants for comment and/or correction?	6
**Domain 3: analysis and findings**		
24. Number of data coders	How many data coders coded the data?	5
25. Description of the coding tree	Did authors provide a description of the coding tree?	5; appendix
26. Derivation of themes	Were themes identified in advance or derived from the data?	5
27. Software	What software, if applicable, was used to manage the data?	5
28. Participant checking	Did participants provide feedback on the findings?	6
29. Quotations presented	Were participant quotations presented to illustrate the themes/findings? Was each quotation identified?	6-9
30. Data and findings consistent	Was there consistency between the data presented and the findings?	6-10
31. Clarity of major themes	Were major themes clearly presented in the findings?	6-10
32. Clarity of minor themes	Is there a description of diverse cases or discussion of minor themes?	9-10

*Tong A, Sainsbury P, Craig J. Consolidated criteria for reporting qualitative research (COREQ): a 32-item checklist for interviews and focus groups. International journal for quality in health care. 2007;19(6):349-57.
